# The Physio-Pathological Role of Group I Metabotropic Glutamate Receptors Expressed by Microglia in Health and Disease with a Focus on Amyotrophic Lateral Sclerosis

**DOI:** 10.3390/ijms24065240

**Published:** 2023-03-09

**Authors:** Matilde Balbi, Giambattista Bonanno, Tiziana Bonifacino, Marco Milanese

**Affiliations:** 1 Department of Pharmacy (DIFAR), University of Genoa, Viale Cembrano 4, 16148 Genova, Italymarco.milanese@unige.it (M.M.); 2 IRCCS Ospedale Policlinico San Martino, Largo Rosanna Benzi 10, 16132 Genoa, Italy; 3 Inter-University Center for the Promotion of the 3Rs Principles in Teaching & Research (Centro 3R), 56122 Pisa, Italy

**Keywords:** microglia, reactive phenotype, group I metabotropic glutamate receptors, mGlu5 receptor, mGlu1 receptor, mGluR5, mGluR1, amyotrophic lateral sclerosis, disease-associated microglia, DAM

## Abstract

Microglia cells are the resident immune cells of the central nervous system. They act as the first-line immune guardians of nervous tissue and central drivers of neuroinflammation. Any homeostatic alteration that can compromise neuron and tissue integrity could activate microglia. Once activated, microglia exhibit highly diverse phenotypes and functions related to either beneficial or harmful consequences. Microglia activation is associated with the release of protective or deleterious cytokines, chemokines, and growth factors that can in turn determine defensive or pathological outcomes. This scenario is complicated by the pathology-related specific phenotypes that microglia can assume, thus leading to the so-called disease-associated microglia phenotypes. Microglia express several receptors that regulate the balance between pro- and anti-inflammatory features, sometimes exerting opposite actions on microglial functions according to specific conditions. In this context, group I metabotropic glutamate receptors (mGluRs) are molecular structures that may contribute to the modulation of the reactive phenotype of microglia cells, and this is worthy of exploration. Here, we summarize the role of group I mGluRs in shaping microglia cells’ phenotype in specific physio-pathological conditions, including some neurodegenerative disorders. A significant section of the review is specifically focused on amyotrophic lateral sclerosis (ALS) since it represents an entirely unexplored topic of research in the field.

## 1. Role of Microglia in the Central Nervous System

Microglia represent the immune cells resident in the central nervous system (CNS) and are, therefore, the primary immune defense of this central area of the human body. Several studies suggest that microglia mainly originate from the embryonic yolk sac, and this evidence was confirmed in rodent models and humans [1,2,3,4,5]. In mice, microglia precursors enter the CNS around day 9.5 of embryonic life (E9.5) through extravascular pathways, as the first cerebral capillaries appear only on day E10 and are soon closed off from the periphery by E13.5, due to the blood–brain barrier’s (BBB) formation. In humans, microglia penetrate the cerebral cortex by gestational week 4.5 (GW4.5); then, a second wave of microglia infiltration penetrates the embryonic brain via the vasculature at GW12–13 [6,7,8,9,10,11,12]. After entering the brain, microglial cells rapidly proliferate [11,13,14,15] and, by GW22, take on a ramified morphology, becoming fully mature by GW35. Early entry and subsequent brain colonization represent actual events in the initial brain development.

Microglia regulate the number of neural precursors, promote neuron survival, and are involved in the phagocytosis of damaged neurons, synaptic pruning, angiogenesis, synaptogenesis, and the maturation of neural circuits [6,11,16,17,18,19,20,21,22,23]. Microglia have a unique genetic signature with respect to the perivascular, meningeal, and choroidal macrophages in the CNS, probably due to macrophages’ presumed hematopoietic origin and developmental processes [24]. Similarly, pre-natal and post-natal microglia differ from adult microglia [25,26]. Microglia play critical physiological roles during CNS development. Resting microglial cells exhibit numerous ramifications that constantly monitor the surrounding microenvironment, thus maintaining CNS homeostasis by phagocytosing cellular debris [27,28]. Microglia play an essential role in synaptic remodeling through synaptic pruning to optimize neurotransmission processes and are directly involved in the formation and reorganization of neural networks and in providing trophic support to mature neurons [12,20,29].

Microglia can sense the CNS environment by monitoring the signaling pathway molecules that guarantee the physiological crosstalk between microglia and neurons, which is fundamental for the maintenance of cerebral homeostasis [12,30,31,32,33]. A minimal variation in the extracellular milieu composition allows microglia to respond by modulating neuronal activity. The mature homeostatic microglia phenotype progresses in a multiple-step process during CNS development and requires continuous instructions from the adult brain [34,35,36,37,38].

Furthermore, microglia are promptly activated in response to CNS stimuli or pathological conditions and undergo massive morphological and functional changes accompanied by rapid clonal proliferation [39,40,41], a process identified as microglia activation. Activated microglia change their morphology from branched to amoeboid, resembling macrophages circulating in the bloodstream, and migrate toward the lesion site [28,42]. Convergence at the injury site occurs in response to signal molecules released by damaged neurons [42]. Activated microglia-induced neurotoxic effects can occur due to the release of cytotoxic molecules, including pro-inflammatory mediators, such as tumor necrosis factor α (TNF-α) or interferon γ (INFγ), and free radicals, superoxide anion, or nitric oxide (NO), and constituents triggering oxidative stress [43]. Under specific conditions, activated microglia acquire a defined anti-inflammatory phenotype, thus releasing neuroprotective factors [44].

Microglia express membrane receptors for several neurotransmitters [45], allowing them to respond to various external stimuli that determine the cell status. Indeed, some neurotransmitters can influence the activation state of microglia, producing changes in membrane potential as well as in the intracellular calcium concentration, causing the release of cytokines and generating cell motility [45,46]. In homeostatic or resting conditions, microglia exhibit the so-called “surveillant” phenotype, characterized by small cell bodies, limited cellular mobility, and extensive and highly mobile branches to control the surrounding environment [28,42]. This quiescent state is commonly identified as neutral, or “M0” [28,47], and characterized by the low expression of surface markers typical of circulating macrophages, i.e., the common lymphocyte antigen (CD45) and major histocompatibility complex class II (MHCII).

As discussed above, microglia are extremely sensitive to changes in the environment, thus being rapidly activated after exposure to specific signals, such as growth factors, neurotransmitters, or cytokines, that indicate the presence of infection, trauma, neuronal damage, or inflammation [48,49]. Moreover, microglia classify pathogens by recognizing damage-associated molecular patterns (DAMPs), receptor patterns of exogenous microorganisms, or endogenous cells involved in the immune response. As for peripheral macrophages, according to the first proposed nomenclature, the microglia activation state includes at least two distinct phenotypes: M1, described as a pro-inflammatory and neurotoxic phenotype, and M2, also known as the “alternative activation phenotype” [50,51,52], with anti-inflammatory and neuroprotective properties. These two phenotypes differently respond to distinct signals from the microenvironment and, in turn, are involved in producing many effector molecules [53], promoting the transcription of genes that activate cellular defense mechanisms, including the release of inflammatory cytokines and chemokines [50,54].

An example of the adaptative response of microglia cells is the exposure to lipopolysaccharide (LPS) or IFN-γ, which, in vitro, converts microglia into an activated detrimental phenotype, and stimulates the release of pro-inflammatory factors, including interleukin-1α (IL-1α), interleukin-1β (IL-1β), interleukin-6 (IL-6), interleukin-12 (IL-12), interleukin-23 (IL-23) cytokines, TNF-α, chemokine (C-C motif) ligand 2 (CCL2), prostaglandin E2 (PGE2), and reactive oxygen species (ROS), through the stimulation of inducible NO synthase (iNOS) [53,55,56]. In contrast, the defensive microglia phenotype is induced by anti-inflammatory cytokines, such as interleukin-4 (IL-4), interleukin-10 (IL-10), or interleukin-13 (IL-13); it suppresses inflammation, promotes the phagocytoses of cell debris, promotes the regeneration of the extracellular matrix, and supports the survival of neurons by releasing protective/trophic factors [52,53,57]. Furthermore, a third microglia activation state, recently defined as the “acquired deactivation phenotype”, represents a different anti-inflammatory phenotype mainly induced by the phagocytosis of apoptotic cells or exposure to anti-inflammatory cytokines, such as IL-10 and transforming growth factor β (TGF-β) [52].

However, this unequivocal classification is inconsistent with the vast repertoire of microglial phenotypes and core functions in different situations, i.e., development, plasticity, ageing, and diseases. As recently reported, considering the coexistence of multiple states, microglia phenotypes occurring in a specific condition should be characterized by more potent analysis tools than those presently applied, such as proteomic, metabolomic, transcriptomic, morphological, and epigenetic ones [58]. Accordingly, a new nomenclature is needed to define the microglia phenotype in each specific physio-pathological environment. This scenario is even more complex since microglia cells can acquire specific activation cellular patterns depending on the pathological environmental conditions in which microglia participate, thus leading to different and peculiar “disease-associated microglia” (DAM) phenotypes [59].

## 2. Glutamate Receptors Expressed in Microglia

The specific activities of microglia likely result from the cell state, regulated by different environmental stimuli, which in turn activate cellular structures, mainly receptors, that act as sensors of external messengers and trigger several intracellular signals with distinct biological functions [46]. The excellent recent literature reports the presence and the role of many microglia-expressed receptor families. Purinergic, serotoninergic, histaminergic, and cannabinoid receptors are the most relevant in tuning the microglia state by regulating their phenotypic characteristics and functions, including proliferation, branch motility, cytokine release, cell migration, and phagocytosis, in physiological [60,61,62,63,64] and pathological conditions, including neurodegenerative diseases [65,66,67,68,69,70,71]. Although supported by limited evidence in the literature, it is worth mentioning that microglia also express GABAergic, cholinergic, adrenergic, and dopaminergic receptors [45,72].

Glial cells, including microglia, widely express glutamatergic receptors, whose activation exerts numerous crucial effects on the glia themselves and glia–neuron interactions in physiological and pathological conditions [73,74]. Glutamate (GLU) is the primary excitatory amino acid neurotransmitter in the brain. Once released at the presynaptic level, it activates post-synaptic α-amino-3-hydroxy-5-methyl-4-isoxazole propionic acid (AMPA), N-methyl D-aspartate (NMDA), and kainate ionotropic receptors to stimulate rapid synaptic transmission [75]. GLU also activates G-protein-coupled metabotropic receptors (mGluRs) with slower signaling transduction kinetics. mGluRs include eight subtypes classified into three groups, termed I, II, and III, based on the sequence homology, signal transduction mechanism, and pharmacological profile [76]. When activated by GLU, mGluRs can generate fine feedback mechanisms at the pre-synaptic level, inhibiting or potentiating the release of GLU itself or other neurotransmitters from heterologous nerve terminals [77,78,79,80]. At the same time, mGluRs regulate critical cellular mechanisms, such as post-synaptic excitatory or inhibitory currents or the activity of glial cells surrounding the synapse, namely astrocytes and microglia [81]. In addition to the numerous actions in glial cells, the activation of mGluRs mediates the interaction between glia and neurons [82,83]. The latter effects are complex and bidirectional, often depending on the implicated mGluR subtypes [83].

Microglia express both ionotropic and metabotropic GLU receptors. These receptors mediate the response to GLU and participate in neuroinflammation and neurodegeneration processes [72]. Microglial NMDA receptors trigger neuroinflammation and neuronal death [84] by also driving pro-inflammatory responses via poly(ADP-ribose) polymerase-1 (PARP-1)/transient receptor potential cation channel subfamily M member 2 (TRMP2) signaling [85]. Thus, the existence of microglial NMDA receptors further offers a link between inflammation and excitotoxicity. Moreover, AMPA and kainate receptor activation triggers microglia reactivity and motor neuron toxicity [86,87,88]. mGluRs in microglia are involved in neuroinflammation [89], acute and chronic neurological disorders, and neurodegenerative diseases [90].

Despite the presence of many mGluR subtypes and their specific functions in finely regulating the cellular processes, in the present review, we focus on the group I mGluRs since they play crucial roles in many physio-pathological situations, as described in the next section. We believe that it is worth exploring their pharmacological or genetic modulation as a potential therapeutic strategy for many traumatic or disease-related conditions.

## 3. Physio-Pathological Role of Group I Metabotropic GLU Receptors Expressed by Microglia

Metabotropic GLU receptors are organized into three groups, termed I, II, and III, overall including eight subtypes (mGlu1–8 receptors) [76]. Group I include mGlu1 and mGlu5 receptors (henceforth reported as mGluR1 or mGluR5), which couple to a Gq protein, resulting in the activation of phospholipase C (PLC), and inositol triphosphate (IP3) and diacylglycerol (DAG) production. Group I receptors are mainly located in the post-synaptic compartment, and their activation increases cellular excitability. Group II, including mGluR2 and mGluR3, and group III, including mGluR4 and mGluR6–8, couple to Gi/Go proteins and inhibit adenylate cyclase (AC) activity and cyclic adenosine monophosphate (AMP) formation. Due to their widespread expression throughout the nervous system and the modulation of the relevant mechanisms that they participate in, mGluRs represent promising therapeutic targets to shape the microglia phenotype [91,92]. Some mGluR ligands are currently under clinical development regarding the treatment of various disorders, such as X Fragile, schizophrenia, Parkinson’s disease (PD), L-dopa-induced dyskinesias, generalized anxiety disorder, and chronic pain [93,94,95].

Microglia cell lines and primary cultures from the cerebral cortex express mGluR5 mRNA and protein [90,96,97]. Although some evidence indicates that quiescent microglia in the healthy brain do not express mGluR5 [98], after spinal cord or head trauma, activated microglia in the vicinity of the lesion significantly express this receptor [90,99]. Conversely, cultured microglia would not express mGluR1 [90,96], but we will present more information below.

Evidence demonstrating the presence of mGluR1 and mGluR5 in microglia has been obtained using selective receptor agonists and antagonists. The expression of the mGluR5a mRNA and the stimulation of calcium signaling by the mGluR1/5 agonist trans-(1S,3R)-1-amino-1,3-cyclopentane dicarboxylic acid (1S,3R-ACPD) took place in cultured microglia, indicating the expression of the mGluR5a variant in these cells [96]. However, Whittemore et al. [100] obtained conflicting results since they found no intracellular calcium signaling modification in microglia stimulated with 1S,3R-ACPD. The reasons for this discrepancy need to be clarified.

Other group I mGluR agonists trigger the activation of PLC in microglia cultures, which leads, again, to the release of calcium and activation of protein kinase C (PKC) [101]. In turn, PKC activation can cause changes in rectifying potassium channel expression and they shape microglia from the ameboid to the ramified phenotype [102]. Signaling downstream group I mGluRs include mitogen-activated protein kinase (MAPK), extracellular signal-regulated kinase 1 (ERK1), and extracellular signal-regulated kinase 2 (ERK2), which are inhibited by selective mGluR5 and mGluR1 antagonists, such as 2-Methyl-6-(phenylethynyl)pyridine (MPEP) and 7-(Hydroxyimino)cyclopropan[b]chromen-1a-carboxylate ethyl ester (CPCCOEt [101,103]). In cultured microglia, the selective activation of mGluR5 by (RS)-2-chloro-5-hidroxyphenylglycine (CHPG), or the combination of the mixed mGluR1/5 agonist [(S)-3,5-Dihydroxyphenylglycine] (DHPG) and the selective mGluR1 antagonist CPCCOEt, attenuated the lipopolysaccharide (LPS) or INFγ-induced activation [97,104], also reducing the accumulation of ROS, the production of TNF-α, and the levels of iNOS with consequent NO release. Moreover, PLC and PKC inhibitors and calcium chelators attenuated the anti-inflammatory events following mGluR5 activation, suggesting that the mGluR5 activation in microglia involves the Gq protein signal transduction pathway [105].

Loane et al. [97] showed that microglia express functional mGluR5, whose activation decreases the release of inflammatory molecules and the impact on neurotoxicity. The inhibition of NADPH oxidase mediated the protective effects of mGluR5 activation in microglia [106], a mechanism of microglia-mediated neurotoxicity common to numerous neurodegenerative diseases [107].

Another molecular pathway linked to the beneficial effects of mGluR5 in microglia is the brain-derived neurotrophic factor/tyrosine-protein kinase B (BDNF/TrKB) cascade [108]. Indeed, the activation of mGluR5 by CHPG protects from oxygen–glucose deprivation (OGD) and reperfusion-induced cytotoxicity, apoptosis, the accumulation of ROS, and the release of inflammatory cytokines in the microglial BV2 cell line [108]. mGluR5 activation also triggers the protein kinase B/glycogen synthase kinase 3β/cAMP-response element binding protein (Akt/GSK-3β/CREB) pathway, resulting in the inhibition of GSK-3β expression, increased phosphorylation of CREB, and reduced expression of inflammation-related genes in microglia cells [109].

Although initially only mRNA coding for mGluR5 was found in microglia cells, and cultured microglia apparently do not express mGluR1, in fact, the mGluR1 subtype appears to be present in this cell population [96], even if less expressed with respect to mGluR5 [105]. In addition to microglia, mGluR1 is also expressed by several cells within the CNS, including neurons, meningeal cells, astrocytes, and T and B cells [96,110], and mGluR1 agonists boost T cell proliferation and promote the activation of the MAPK signaling cascade, increasing inflammation [111]. Additionally, mGluR1 immunoreactivity has been reported in a subset of the microglia/macrophage cell lineage in human multiple sclerosis (MS) lesions [112]. Therefore, mGluR1 antagonists might also have multiple therapeutic applications.

### 3.1. Role of Group I Metabotropic GLU Receptors in Microglia-Mediated Neuroinflammation

The modulation of microglia’s reactive phenotype and inflammation state has been demonstrated in vitro and predominantly involves the mGluR5 subtype, thus highlighting this receptor as a potential pharmacological target. Byrne et al. demonstrated that mGluR5 stimulation decreased neuroinflammation in vivo after spinal cord injury [105]. Moreover, a single-dose treatment with CHPG significantly improved functional recovery, modulated neuroinflammation, and limited lesion progression after experimental traumatic brain injury, most likely through mGluR5 activation [113]. Fibrinogen-mediated microglial activation was downregulated by the direct activation of mGluR5, providing neuronal protection [114].

Based on the above evidence, new molecules, such as positive allosteric modulators (PAMs), have been developed [115] and the in vivo application has highlighted the importance of reducing inflammation in different pathological conditions. In this context, Loane et al. [116] showed that treatment with mGluR5 PAM VU0360172 significantly reduced neurodegeneration in the mouse hippocampus and improved motor function recovery after controlled cortical impact. These effects were mediated by mGluR5, which reduced cluster of differentiation 68 (CD68) and NADPH oxidase 2 (NOX2) expression and suppressed pro-inflammatory signaling pathways in activated microglia. In addition, VU0360172 treatment shifted the balance between pro-inflammatory and anti-inflammatory microglia activation states toward a protective and pro-reparative phenotype [116]. The pharmacological stimulation of mGluR5 also attenuated the production and release of TNF-α in a Theiler’s murine encephalomyelitis virus (TMEV)-induced model of seizures/epilepsy [117]. The protective role of mGluR5 was recently demonstrated by Carvalho et al. as the specific receptor whose ablation accelerated age-related neuroinflammation and neurodegeneration in a Huntington’s disease mouse model [118]. The α-synuclein-induced microglia inflammation, mimicking PD, was also reduced by the activation of mGluR5 [119], possibly reducing the inflammatory component of this pathology [120]. It is also worth noting that mGluR5 and Toll-like receptor 4 (TLR4), two critical receptors for microglia activation [121], coexist in microglia and that LPS triggers the downregulation of the mGluR5 gene expression by TLR4 activation [122].

### 3.2. Group I Metabotropic GLU Receptor-Mediated Modulation of Microglia-Released Extracellular Vesicles

Microglia affect neuronal activities by releasing various mediators. Nonetheless, additional intercellular communication mechanisms have been recently described, such as those mediated by microvesicles (MVs), macrovesicles, and exosomes [123,124,125]. The extracellular vesicles released by microglia significantly differ in size, content, and intracellular organelle origin. They are formed either constitutively or, as in the case of microglia, under stimulating conditions, including the activation of receptors expressed at the cell membrane [126,127,128,129]. Vesicles from microglia diffuse into the extracellular space, shuttling toward recipient cells, cytokines, miRNAs, lipids, and other factors [126,130,131,132]. Although the precise kinetics of this interaction are only partially known, the release of vesicles by microglia has been clearly described [124,133,134,135]. Released extracellular vesicles mediate neurotoxicity mainly by transferring pro-inflammatory cytokines [130,136] or even modulating protein aggregation, as in the case of β-amyloid [137]. Furthermore, extracellular vesicles were increased in the human and rodent cerebrospinal fluid during cerebral inflammation, supporting their role in the spread of the inflammatory process [135,138] and in neurodegenerative diseases [139,140,141,142].

Indeed, we need to learn more about the role of group I mGluR activation or blockade in the modulation of the release of extracellular vesicles and their cargo from microglia cells. Activating the P2X7 receptor triggers the release of MVs from microglia, and the same occurs after the stimulation of mGluR5 by CHPG [143]. Of note, LPS-activated microglia blunted the mGluR5 effect, possibly indicating the downregulation of the receptor after LPS stimulation. MVs produced by microglia exposed to CHPG significantly increased the rotenone-induced MN neurotoxicity, suggesting a pivotal role of mGluR5 in regulating the cargo rather than the number of released MVs. Interestingly, miR146a was upregulated in microglia cells by pro-inflammatory signals [144,145] and highly expressed in the MV cargo after microglial mGluR5 activation [143].

## 4. Role of Group I mGluRs Expressed by Microglia Cells in Specific Traumatic and Pathological Conditions

**Figure 1:**
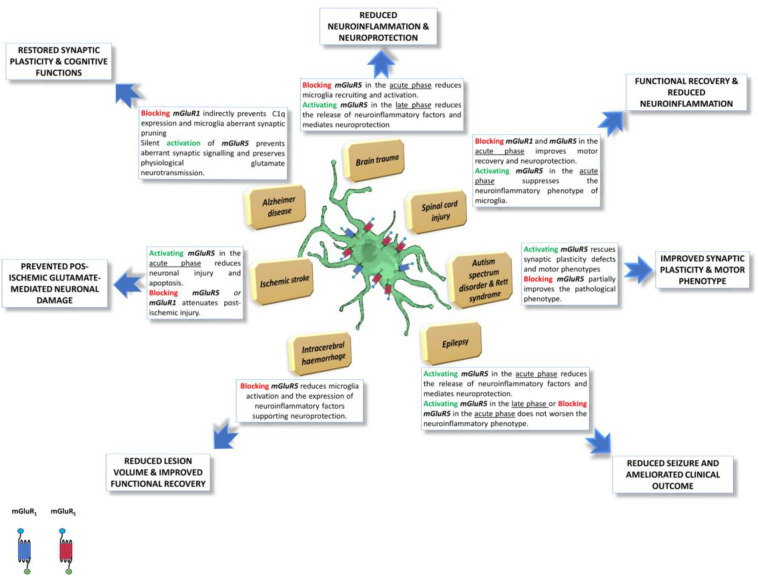
Schematic representation of microglia-expressed group I mGluRs effects, in different pathological conditions. Group I mGluRs are key regulators in different pathological conditions, directly or indirectly influencing the microglia cell activation state. Pharmacological or genetic modulation of Group I mGluRs, also expressed by microglia cells, can affect the disease course, with often dual or biphasic effects, depending on the time window of intervention and duration of the treatment, overall being a promising tool to develop new pharmacological drugs. The figure was partly generated using Servier Medical Art, provided by Servier, licensed under a Creative Commons Attribution 3.0 unreported license “https://creativecommons.org/licenses/by/3.0/ (accessed on 19 January 2023)”.

Based on the evidence in the literature, the group I mGluRs represent key regulators in different pathological conditions, directly or indirectly influencing the microglial activation state. As schematically illustrated in Figure 1, the following section aims to elucidate the effect triggered by the positive or negative modulation of mGluR1 and mGluR5, also expressed by microglia cells, focusing on their potential exploitation as druggable targets for therapeutic interventions.

### 4.1. Brain Trauma

Traumatic brain injury (TBI) is a common and often life-threatening clinical condition [146,147], that begins within seconds or minutes after the traumatic insult and can last for days, weeks, and potentially months or years [148]. After an injury, microglia cells undergo marked morphological and behavioral changes, switching from branched to amoeboid cells, followed by proliferation and migration to the lesion site [42].

Evidence in the literature highlights a dual role of mGluRs in TBI. In the acute phase, Yang et al. [149] demonstrated that the genetic ablation of mGluR5 slowed down the progression of cerebral inflammation by reducing the activation of circulating immune cells and their massive infiltration through the BBB in the traumatic area, thereby decreasing the onset of the neuroinflammatory state and improving the clinical scenario. Mechanistically, the mGluR5 absence blocks the signaling pathway mediated by PKC and causes the inhibition of chemokine expression [150]. In the delayed phase, stimulation of microglial mGluR5 beneficially modulates the neuroinflammation-linked microglia-mediated neurodegenerative mechanisms. Moreover, Byrnes et al. [113] demonstrated that microglia-expressed mGluR5 is more reactive to CHPG after TBI, thus inhibiting the persistent post-injury neuroinflammation and reducing it. Indeed, treatment with CHPG significantly improved long-term sensorimotor and cognitive recovery while reducing the number of activated microglia cells, the persistent post-injury neuroinflammation, and the tissue loss after trauma. The beneficial effects can be attributed to the activation of mGluR5 since the administration of a selective mGluR5 antagonist blocked this effect. However, further investigation is needed to better explain how the selective modulation of mGluR5 expressed by microglia cells can directly impact brain injury.

### 4.2. Spinal Cord Injury

Spinal cord injury (SCI) is a severe pathological condition leading to cell damage and death [151]. During SCI, CNS-resident microglia become rapidly reactive and release cytokines, leukotrienes, prostaglandins, NO, and superoxide radicals that produce neurotoxic and neurodegenerative effects [152].

Although the microglia-expressed group I mGluRs play a crucial role in shaping inflammation in SCI, we need to learn more about these potential targets. The expression of mGluRs changes after SCI [153], which most likely also occurs in microglia. In particular, mGluR1 expression increases with time following injury, while mGluR5 and mGluR2/3 decrease acutely or chronically, respectively. The opposite effect of mGluR1 and mGluR2/3/5 could contribute to balancing the clinical outcome after SCI. In 2002, Mills et al. [154] showed that a single treatment with the mGluR1 selective antagonist LY367385 improved locomotor scores and attenuated the development of mechanical allodynia. On the other hand, treatment with the selective mGluR5 antagonist MPEP attenuated the development of thermal hyperalgesia, although it did not affect locomotion and mechanical allodynia. The positive effects of the two antagonists suggest distinct acute pathophysiological roles for the two group I mGluR subtypes after SCI. However, further investigation is needed into the direct involvement of mGluR1 and mGluR5 expressed by microglia and the effect triggered by their pharmacological blockade.

Subsequently, the results published by Byrnes et al. [155] provided further evidence, showing that mGluR5 receptor agonists could significantly improve histopathological and functional outcomes after SCI. As an in vitro proof-of-concept, the authors showed that the activation of spinal cord microglia by stimulating mGluR5 with CHPG suppressed the expression of inflammatory markers, such as iNOS, NO, galectin-3, and TNF-α, and reduced microglia neurotoxicity. The above evidence suggests that the pharmacological activation of mGluR5 after SCI may contribute to the protective effects via an anti-inflammatory mechanism enacted by microglia cells. However, we cannot exclude the simultaneous in vivo effects of CHPG on circulating macrophages and other CNS cells, such as astrocytes, that clearly express high mGluR5 levels, especially under pathological conditions.

It is worth recalling that the influence of microglia and macrophages in SCI is a controversial topic. The type of treatment, acute or chronic, with pharmacological tools acting on group I mGluRs, can lead to neuroprotective or neurotoxic effects. Indeed, the differential activation of microglia and macrophages at different stages after SCI can cause the loss or preservation of the tissue affected by the trauma [156], thus indicating that the neuroprotective effect of microglia modulation after SCI is closely related to the pathological timing. The anti-inflammatory outcomes indicate that mGluR5 activation can have multiple neuroprotective effects, mainly during the early phase after SCI. Notably, as mGluR5 is also expressed by neurons, oligodendrocytes, and astrocytes, selective agonists can positively act in vivo as multimodal drugs [90].

### 4.3. Autism Spectrum Disorder

Autism spectrum disorders (ASDs) are severe conditions affecting childhood neurological development with a multifactorial and polygenic etiology [157]. Zantomio and co-Authors [158] showed the presence of glutamatergic signaling alterations, with particular attention to the mGluR5 receptor and the downstream pathways.

mGluR knockout mice and syndromic and non-syndromic forms of ASD were studied, with a specific incidence in X fragile and Rett’s syndrome [159,160].

In 2009, Blaylock et al. introduced the term “immunoexcitotoxicity” to describe neuronal damage resulting from microglia activation [161]. The increase in the microglia cell number in the fronto-insular, visual, and dorsolateral prefrontal cortex [162,163] in the post-mortem brains of ASD patients, and the presence of activated microglia in the cerebellum [164], manifests a chronic inflammation status sustained by pathologically activated microglia cells.

The link between neuroinflammation and ASD is also supported by the increase in pro-inflammatory cytokines in the blood and CSF [165,166,167]. mGluR5 represents a central actor in the synapse alteration and neuroinflammation observed in ASD. Moreover, the downstream mGluR5 signaling pathways are closely related to many rare gene variants associated with the pathogenesis of ASD, such as the coding elements *SHANK1*, *SHANK2*, and *SHANK3* [168,169,170].

The subtle correlation between ASD, microglia, and mGluRs is supported by the hypothesis that mGluR5 and the Fragile X Messenger Ribonucleoprotein 1 (FMRP) may exert opposite actions on neuronal plasticity. However, the direct role of mGluR5 in maintaining microglia homeostasis during development has yet to be fully elucidated. Chana et al. demonstrated that the decreased expression of mGluR5 represents a key pathophysiological hallmark in ASD since it regulates the microglia cell number and synaptic pruning during development, with the preservation of appropriate connectivity for normal brain functioning [171]. The *Grm*5 gene expression was significantly decreased in ASD and associated with increased pro-inflammatory markers. These studies provide evidence of a connection between mGluR5 signaling, microglia cells, and a syndromic form of ASD in the X Fragile mouse model. Nevertheless, further investigation is needed to fully elucidate the role of microglia and mGluRs in this context.

### 4.4. Rett’s Syndrome

Another neuropathological condition characterized by a severe cognitive deficit often associated with an ASD context is Rett’s syndrome. An alteration in the regulatory role of the methyl-CpG binding protein 2 (MeCP2) gene, indirectly linked to mGluR5, is part of the disease etiology [172]. In Rett’s syndrome, mGluR5 signaling is reduced, accompanied by immune dysregulation [160,173]. MeCP2 phosphorylation is required to modulate synaptic scaling via mGluR5 activation [160]. Indeed, microglial cells lacking MeCP2 showed increased neurotoxicity supported by the release of neurotoxic factors without altering their morphology and the proliferation rate [174]. Interestingly, when MeCP2 was restored only in microglia via bone marrow transplantation, both microglial dysfunction and Rett’s syndrome behavioral symptoms were significantly ameliorated [175]. This finding indicates that the microglia-limited MeCP2 deficit is sufficient to produce the symptoms of Rett’s syndrome and increases the importance of microglia in non-syndromic ASD forms, thus providing evidence that manipulating the immune response can have significant therapeutic potential.

Further results from Gogliotti et al. demonstrated that the mGluR5 PAM VU0462807 could rescue synaptic plasticity and motor defects in a mouse model of Rett’s syndrome [176]. Conversely, the chronic treatment of MeCP2 KO mice with the mGluR5 negative allosteric modulator (NAM) 2-chloro-4-((2,5-dimethyl-1-(4-(trifluoromethoxy) phenyl)-1H-imidazol-4-yl)ethinyl)pyridine (CTEP) partially improved the pathological phenotypes of mice and reduced the upregulated MeCP2-linked gene level, establishing a potential mechanistic link between MeCP2-dependent transcription repression and the mGluR5 pharmacological modulation [177]. Therefore, the microglia-expressed mGluR5 could represent a challenging target for the modulation of Rett’s syndrome and other cognitive deficits.

### 4.5. Epilepsy

Several studies exploiting Theiler’s murine encephalomyelitis virus (TMEV) and other epilepsy rodent models demonstrated the importance of microglia neuroinflammation in the development of seizures [178,179,180,181,182,183]. TMEV infection causes the infiltration of circulating immune cells in the brain [184] and the activation of microglia [178,179].

Interestingly, the stimulation of mGluR5 by the PAM VU0360172 reduced the production of microglial L-6 and TNF-α and generated neuroprotective effects [97,185]. Short-term treatment with VU0360172, from days 0 to 3 after infection, reduced seizures, while long-term treatment, up to 8 days after infection, did not further reduce seizures in TMEV-infected mice. These experiments showed that the stimulation of mGluR5 suppresses the production of TNF-α at seizure onset; thus, one possible mechanism supporting the improvement in TMEV-infected mouse clinical outcomes after short-term treatment is the reduced amount of TNF-α released by microglia and macrophages. Interestingly, treating TMEV-infected mice with the mGluR5 NAM 3-((2-Methyl-4-thiazolyl)ethynyl)pyridine (MTEP) did not worsen seizures, probably because blocking mGluR5 did not increase the TNF-α levels, or the further production of TNF-α by microglia or macrophages did not exacerbate the seizures.

These studies show that inflammation is essential in the development of seizures and epilepsy, which do not depend solely on viral infection, and again supports the role of microglia and the importance of modulating mGluR5 signaling within an early, critical therapeutic window.

### 4.6. Intracerebral Hemorrhage

Hemorrhage in the brain parenchyma, here termed intracerebral hemorrhage (ICH), often has devastating consequences [186], with long-term neuronal damage and neurological deficits [187,188].

After ICH, the activation and aggregation of microglia are the most common expressions of the immune response. They are among the earliest and most significant events in the maintenance of post-ICH damage [189,190]. Resting microglia increase the expression of mGluR5 significantly only after brain injury [99,191]. The upregulation of mGluR5 induced by ICH promotes the activation of microglia, facilitating the release of the inflammatory cytokines TNF-α and IL-6 close to the lesion site. Therefore, reducing the reactive microglia by in vivo pharmacologically inhibiting mGluR5 can preserve neuronal death and promote functional recovery after stroke. In line with this, several observations support the therapeutic potential of mGluR5 antagonists, which, via reduced cytoplasmic calcium mobilization [121], can modulate microglia’s activation state and, consequently, reduce neurodegeneration and neuronal apoptosis [192,193,194,195].

Very recently, Rahman et al. confirmed this occurrence, showing that reactive microglia highly expressed mGluR5 after ICH and that the in vivo inhibition of mGluR5 by MTEP resulted in attenuated microglial activation and reduced cytokine release [196]. Notably, this effect translated into a marked reduction in cell apoptosis and neurodegeneration, a significant decrease in the lesion volume, and improved functional recovery [196].

Although previous studies reported that mGluR5 stimulation reduces microglia activation and the associated inflammatory response [105,116,185], the above results are consistent with contrasting evidence that mGluR5 blockade reduced microglial activation [121,197,198,199]. The different experimental models adopted and the diverse time windows of the pharmacological treatments might partly explain these discrepancies [197].

Overall, there is a broad consensus that after ICH, mGluR5 attenuation decreases the activated microglia, inflammatory response, neuronal death, and neurological deficit. Therefore, mGluR5 blockade may represent a potential strategy to treat ICH, and microglia indeed represent a potential target for mGluR ligands to modulate the microglia-activated phenotype in ICH. However, further pre-clinical evidence is needed to confirm this hypothesis and clarify the contrasting results in the literature.

### 4.7. Ischemic Stroke

Ischemia caused by stroke is one of the leading causes of mortality and long-term disability worldwide [200]. The modulation of the GLU-mediated apoptotic pathway is one of the most promising strategies for the development of new drugs. Indeed, excessive GLU release after ischemic injury triggers an excitotoxic cascade, which leads to receptor-mediated neuronal cell death [201,202]. Ischemic injury consists of three phases [203]. Whereas neuronal necrosis occurs early after stroke, delayed neuronal death emerges several hours, days, or weeks after the primary damage due to apoptosis [204]. During the subacute phase of ischemic stroke (24–72 h after onset), vasogenic oedema begins [205]. Weeks after ischemia onset, the chronic phase leads to additional tissue damage and may result in delayed neurodegeneration triggered by oxidative stress and immune activation.

Microglia modify the expression of GLU receptors only after priming conditions [206]. During brain hypoxia and ischemia, microglia release elevated amounts of GLU and other factors that, in turn, influence GLU homeostasis [207], thus directly contributing to the excitotoxic injury.

Experimental evidence indicates that mGluR5 play different roles in modelling cerebral ischemia conditions. Interestingly, the mGluR5 mRNA level significantly increased in the middle cerebral artery occlusion (MCAO) model of ischemia [208]. Since microglia are activated after ischemic stroke and mGluRs may represent a potential target for modulating the microglia’s detrimental phenotype, the pharmacological intervention of group I mGluRs may provide an alternative approach to reducing GLU-mediated microglia activation and neuronal cell death after ischemic stroke. A research report by Bao et al. highlighted that MPEP and CHPG proved a therapeutic potential for treating stroke when acutely administered after focal cerebral ischemia by attenuating apoptotic cell death, although they, respectively, inhibit and activate the receptor [198]. Confirming the role of mGluR5 in ischemic stroke, the pre-ischemic reduction in mGluR5 mRNA levels by exercise training was demonstrated to produce ischemic tolerance in a rat model of MCAO through the PKC-α-GLT-1-GLU interconnected pathways [208,209]. Recently, Cavallo et al. showed that the mGluR5 PAM VU0092273 significantly reduced in vitro the hippocampal neuron injury in the OGD model of ischemia via the PI3K/Akt pathway and the molecular switch of AMPA receptors that indirectly reduce the Ca^2+^ influx [210]. The same group previously provided a different viewpoint when studying the neuroprotective mechanism enacted by mGluR1 antagonists, predicting that the pharmacological blockade indirectly attenuates post-ischemic injury by enhancing the GABA-mediated neurotransmission, which differs from the pathways reported when activating or blocking mGluR5 in microglial cells [211].

Considering the above studies and the fact that microglia express both mGluR1 and mGluR5, the beneficial effects could result from a complex interplay of factors that indeed involve microglia, since they actively contribute to the pathological scenario of ischemic stroke. In turn, the pharmacological modulation of mGluR1 and mGluR5 provides an exciting perspective for new therapeutic interventions. However, dissecting the contributions of the two microglial receptors and determining which would be the most efficacious for clinical application remains challenging.

### 4.8. Alzheimer’s Disease

Alzheimer’s disease involves extensive neuroinflammation, neuron and synapse loss, and memory impairment [212]. Microglia play a central role in inducing and maintaining synaptic plasticity and connections, via synaptic pruning [213]. On the other hand, they can also affect synaptic efficiency in neurological disorders, as demonstrated in transgenic AD mouse models [72,214]. The molecular mechanism of this aspect is unknown, although β-amyloid induced microglia activation and neuroinflammation, enhancing the expression of neuroligin 1 transcription repressors, thus reducing GLU synapses in the hippocampus [215]. Altered microglia–neuron interaction impairs synaptic pruning and the correct brain network development, which are associated with social interaction deficits and altered behavioral phenotypes in rodents [216].

FMRP is an abundant mRNA-binding protein in the brain, that modulates the transport and local translation of synaptic mRNA [217,218], and the increased expression of FMRP has been recently reported in a transgenic mouse model of AD [219]. The upregulation of mGluR1 induces FMRP dephosphorylation and facilitates the local translation of synaptic complement component 1q (C1q) mRNA, the initiator of the classical complement activation pathway promoting microglia-operated synaptic pruning [220,221], consequently increasing the phagocytosis of hippocampal glutamatergic synapses and contributing to cognitive dysfunction in AD rodent models [222]. Accordingly, inhibiting C1q or blocking the microglial complement receptor CR3 reduced the microglia-operated synapse phagocytosis and synaptic loss in the early stage of the disease [223]. Indeed, indirect suppression of p-FMRP de-phosphorylation by inhibiting the mGluR1 transduction signals decreases the expression of synaptic C1q and microglial phagocytosis, favoring the recovery of GLU transmission and cognitive abilities, in rats treated with amyloid fragments [224].

On the other hand, Spurrier et al. recently demonstrated that treatment with the mGluR5 silent allosteric modulator BMS-98492 prevents β-amyloid oligomer-induced aberrant synaptic signaling while preserving the physiological GLU response and restoring synaptic density in AD mouse models [225].

To conclude, the key role of microglia cells in AD, leading to the non-physiological activation of synaptic pruning processes and contributing to a progressive cognitive deficit, is now clear, but the basis for this immune-mediated synaptic attack remains to be elucidated. In this scenario, mGluR1 is pathologically upregulated in microglia and can participate in increased complement-mediated synaptic phagocytosis. However, as depicted in many other traumatic or pathological conditions, the direct role of microglia-expressed group I mGluRs and their involvement in C1q-mediated synaptic phagocytosis in AD has yet to be clarified.

## 5. Microglia and Amyotrophic Lateral Sclerosis

### 5.1. The Multifactorial and Multicellular Facets of Amyotrophic Lateral Sclerosis

Amyotrophic lateral sclerosis (ALS) is a fatal neurodegenerative disease characterized by the progressive loss of upper and lower motor neurons (MNs). In the early phase, symptoms are muscle weakness, followed by a gradual loss of muscle control and contraction, atrophy, and paralysis, which lead to death by respiratory failure [226]. Around 90% of cases of ALS are sporadic or unrelated to a specific etiological or hereditary genetic cause. However, 10% of patients are familial, attributable to specific transmissible genetic mutations [227,228]. The identification of more than 40 genes mutated in ALS, affecting numerous cellular functions, has allowed the generation of different animal models useful for pre-clinical research [229]. Mutations in the superoxide dismutase 1 (SOD1) enzyme were the first identified [230] and SOD1-mutated rodents are still the most used models since they recapitulate the ALS pathology better than others. The most recently discovered and frequent ALS-related mutation relates to a repeated hexanucleotide expansion (GGGGCC) in the C9orf72 gene [231,232]. Many other gene mutations have been identified over the years linked to ALS, the most relevant being fused in sarcoma (FUS) protein [233] and binding TAR-DNA-43 (TDP-43) protein [234] mutations.

At present, the approved therapeutic drugs are riluzole, edaravone, and tofersen. Riluzole was proposed to reduce glutamate neurotransmission but only prolongs the lifespan by 2–3 months [235,236]. Edaravone, which reduces oxidative stress, slightly improves motor function scores in patients but only when administered in the early stages of the disease [237,238]. The most recent, tofersen, is an anti-sense oligonucleotide inhibiting SOD1 synthesis. A recent phase 3 clinical trial demonstrated that by initiating the administration of the anti-sense oligonucleotide in early symptomatic patients carrying SOD1 mutations, the drug effectively slowed the decline in clinical and respiratory function and increased muscle strength, improving the quality of life in SOD1-mutated familial patients [239].

Although the initial ALS approach was mainly focused on neurons, growing and unequivocal evidence indicates that non-neuronal cells also play a key role in the pathogenesis of ALS, thus contributing to the definition of ALS as a non-cell-autonomous disease [240,241,242,243,244,245]. Glial cells, such as astrocytes and microglia, can participate in the local inflammatory response with peripheral lymphocytes and macrophages. They acquire a reactive phenotype, migrate to the lesion site, proliferate, and secrete pro-inflammatory and neurotoxic mediators [246,247,248,249,250,251,252,253,254]. Glial activation modifies the expression of a wide range of soluble molecules, such as cytokines and chemokines, DAMPs, reactive nitrogen species (RNS), and ROS, giving rise to profound changes in fundamental aspects of the interactions between glia and neurons [255].

Microglia become reactive before the symptomatic phase of the disease [52,256], concomitantly with the first loss of neuromuscular junctions [257] and MNs [258]. The SOD1^G93A^ mouse model of ALS demonstrated that the disease onset is associated with microglial activation and TNF-α, IL-6, and IL-1β production, suggesting that the SOD1 mutant protein may trigger a pathogenic response in microglia [259]. On the other hand, microglia can also play a protective role in ALS by secreting anti-inflammatory factors, such as IL-4 and IL-10, and growth factors, thus achieving a crucial balance between the pathogenic and protective phenotypes [260,261]. Likewise, astrocytes and oligodendrocytes represent essential cells in maintaining CNS homeostasis and axonal and neuronal integrity; nevertheless, they undergo functional and molecular alterations in ALS, thus sustaining the degeneration and death of MNs [262,263,264].

### 5.2. The Dual Role of Microglia in Amyotrophic Lateral Sclerosis

Several in vivo studies have shown that circulating microglia cells increase during disease progression and change their activation state [240]; thus, using the former nomenclature, they have been extensively classified as M1- (pro-inflammatory) or M2 (anti-inflammatory)-like phenotypes in ALS [260,265,266]. The microglia phenotype, activated in response to different microenvironmental signals, always includes a balanced ratio between the pro-inflammatory and the anti-inflammatory phenotypes [267,268,269,270], involving the production of different effector molecules [53].

Interestingly, mutant SOD1^G93A^ microglia in ALS differ from wtSOD1 lipopolysaccharide (LPS)-activated microglia and M1/M2 macrophages, defining an ALS-specific phenotype [268] and confirming that the M1/M2 paradigm is an oversimplification of the genuine status, in line with the actual consensus [58,271]. The different microglial cell phenotypes in ALS are mainly based on the characterization of their morphology. The cells in the first phase of the disease, defined as “supervisor” microglia, are characterized by short and poorly branched processes and show an anti-inflammatory profile and overexpression of interleukin IL-10 [272]. In later phases of the disease, microglia show large cell bodies with short and dense processes [273]. Moreover, in the spinal cord of the SOD1^G93A^ mouse, the expression of anti-inflammatory phenotype markers was strongly reduced during the pre-symptomatic phases, while it became more evident in the late symptomatic stage of the disease only [260,274].

Reactive microglia appear hyper-activated in ALS [275]. The toxic activity may be attributed to intracellular mutated proteins, such as TDP-43 and SOD1 [248,267,276]. In particular, the expression of mutated SOD1 (mSOD1) in microglia contributes to the phenotype shift during disease progression. Accordingly, mSOD1-expressing microglia exhibit neuroprotective features during the early phase, improving neuronal survival, while, in the late stage of the disease, mSOD1 microglia exhibit neurotoxic activity [267]. NF-kB, the primary regulator of neuroinflammation, whose expression can be triggered by mutant proteins, plays a central role in the activation of microglia [277]. Upregulation of NF-kB in WT microglia induces gliosis and indirect MN death, both in vitro and in vivo. In contrast, the downregulation of NF-kB in microglia protects MNs from death in vitro and prolongs survival in ALS-affected mice by shifting microglia to anti-inflammatory activity. These data suggest that microglia induce MN death through the activation of the classic NF-kB pathway, which might therefore represent an excellent target in the therapeutic strategies for ALS [277,278]. Several findings indicate that, during ALS progression, microglia cells do not undergo a stage-dependent transition [268]; instead, they show the coexistence of the different phenotypes, thus producing a peculiar functional profile as a complex outcome of multiple regulation factors. It will be very intriguing to further explore the modulatory effect exerted by group I mGluRs on the NF-kB pathway, thus potentially unveiling a pharmacological alternative to the genetic manipulation aimed at modifying microglia cells’ phenotypes.

### 5.3. Glutamatergic Neurotransmission and Microglial Group I Metabotropic Glutamate Receptors in Amyotrophic Lateral Sclerosis

The excitotoxic role of glutamate was one of the first etiopathological mechanisms studied in ALS [279,280,281,282,283]. In line with this, riluzole was, for a long time, the only drug approved for ALS, which modestly improves the disease course through the unspecific inhibition of glutamatergic neurotransmission [284].

Microglia express ionotropic and metabotropic receptors mediating the response to glutamate and participating in neuroinflammation and neurodegeneration [72,83]. In ALS, MNs are damaged in the motor cortex, brain stem, and spinal cord. This damage is also supported by the selective dysfunction of astrocyte glutamate reuptake, further contributing to excitotoxicity and MN death [285]. In 2004, Zhao et al. investigated the effects of primary mouse microglia cells activated by LPS or ALS patients’ IgG immune complexes on MN survival. Microglia indirectly damaged MNs by increasing their susceptibility to glutamate through a reduction in astrocyte reuptake [86]. On the other hand, the release of TNF-α induced by activated microglia produced the significant release of autocrine glutamate, mainly through gap junctions and supported by the upregulation of glutaminase, which generates a direct excitotoxic effect toward MN [286]. The link between microglia and glutamate was further confirmed by the ability of riluzole to modulate the activation of primary rat microglia cell cultures by decreasing the release of LPS-induced pro-inflammatory markers and increasing the production of neuroprotective factors, such as IL-4; thus, this suggests that riluzole could be neuroprotective in ALS via microglia-mediated mechanisms [287].

The release of glutamate by activated microglia occurs mainly by exploiting the cystine/glutamate antiporter (xCT/Slc7a11), the expression of which is increased during disease progression and associated with enhanced inflammation, in microglia cells from the post-mortem spinal cord tissue of ALS patients [288]. Instead, xCT/Slc7a11 is not expressed in MNs [288]. Mesci et al. demonstrated that the genetic deletion of xCT/Slc7a11 in mice decreased the microglia-associated pro-inflammatory factors, such as NO, TNF-α, and IL-6, while increasing the expression of the neuroprotective marker chitinase-like protein 3 (Chil3, known also as Ym1). Surprisingly, the deletion of xCT/Slc7a11 in mutated SOD1G37R ALS mice anticipated the onset but significantly slowed down the progression of the disease, consistent with the dual role of microglia and glutamate’s downstream effects during disease progression [288].

Differing from other pathological or traumatic conditions, the involvement of group I mGluRs in the modulation of the microglia phenotype in ALS is poorly documented. Nevertheless, mGluR1, mGluR5, and microglia cells are indeed a promising target for ALS treatment and other neurodegenerative diseases in which neuroinflammation plays a pivotal role [73,90,92,270]. In ALS patients, mGluR1 and mGluR5 mRNAs are abundantly expressed in the dorsal horn rather than in the ventral horn of the spinal cord. Of note, spared MNs express abundant mGluR5, while vulnerable MNs do not [289]. Aronica et al. showed that mGluR1 and mGluR5 were highly represented in neuronal cells throughout the human spinal cord, with mGluR1 having high expression in ventral horn neurons. In contrast, intense mGluR5 immunoreactivity was observed in the dorsal horns [290]. This different CNS area- and cell-specific expression of group I mGluRs highlights an intriguing clue possibly linked to the selective vulnerability of MNs in ALS [291,292,293,294]. Regarding glial cells, only sparse astrocytes showed weak to moderate staining for mGluR1 and mGluR5 in the spinal cords of healthy patients [290,295]. In ALS patients, the mGluR1 and mGluR5 immunolabeling intensity increased in cells with an astroglia morphology in the grey and white matter. At the same time, their expression in neurons was comparable to that observed in healthy subjects [290,295].

Although the role of mGluR5 in regulating astrocyte function and their neurotoxic phenotype during ALS progression was largely investigated [296,297,298,299,300,301], there is only one paper describing mGluRs affecting microglia functionality [122]. Berger et al. examined in vitro the modulation of mGluRs expressed by microglial cells in two distinct models of inflammatory conditions, including microglia cell cultures obtained from rats expressing the SOD1^G93A^ ALS-linked mutation. As expected, SOD1^G93A^ microglia were characterized by increased neuroinflammation and enhanced reactivity. The results showed that the mGluR5 mRNA was upregulated in microglia cell cultures prepared from the brains of neonatal SOD1^G93A^ rats. Interestingly, the exposure to LPS, mimicking an inflammatory environment, increased mGluR3 and decreased mGluR5 gene expression in both SOD1^G93A^ and wtSOD1 microglia [122]. This evidence indicates that an inflammatory environment may trigger the opposite regulation of mGluR subtype gene expression. These events seem particularly robust in SOD1^G93A^ microglia cultures. Thus, it will be crucial to consider the possible cell-specific receptor expression and localization when considering these therapeutic targets in ALS.

More recently, Wang et al. developed radiotracers that selectively target mGluR5 [302], allowing their characterization, distribution, and functional binding properties by in vivo micro-PET in different experimental animal models, including SOD1^G93A^ mice [303,304]. Exploiting this technique, Brownell et al. described the expression pattern and the effects obtained by the modulation of mGluR5 during progressive degeneration in ALS mice carrying the SOD1^G93A^ mutation [305]. In detail, concomitantly using specific ligands for mGluR5 ([18F]FPEB) and activated microglia cells ([11C]PBR 28) during the inflammatory response, they evidenced that inflammation and mGluR5 expression, colocalizing with the IBA1-positive microglia, were enhanced in the hippocampus, striatum, frontal cortex, and spinal cord of SOD1^G93A^ mice. These data highlighted the role of GLU in promoting the inflammatory response in ALS, through the activation of mGluR5, expressed by microglia cells. Of note, the enhanced expression of mGluR5 in the brains of SOD1^G93A^ mice couples with the inflammation observed in ALS patients by using the same [11C]PBR28 ligand [306].

A significant contribution to our understanding of the potential role of group I mGluRs in modulating the disease progression and microglia reactivity in ALS comes from our research group. Using the SOD1^G93A^ mouse model, we first demonstrated that the expression and function of mGluR1 and mGluR5 were enhanced at glutamatergic synapses in the spinal cord at the early pre-symptomatic and late symptomatic stages of the disease [78,79]. These alterations could further exacerbate the excessive glutamatergic neurotransmission previously demonstrated in the spinal cords of SOD1^G93A^ mice [281,283,307]. In subsequent studies, genetically halving mGluR1 and mGluR5, or ablating mGluR5, significantly ameliorated disease progression and survival probability in SOD^G93A^ mice [308,309,310], and produced a reduction in astrogliosis and microgliosis, always accompanied by positive outcomes for the ALS phenotype. In vivo, the beneficial effects can hardly be ascribed to a specific cell subtype; however, we postulated that the observed modulation of reactive astrocytes and microglia could represent a potential contribution to the improved MN survival [308,309,310]. Unfortunately, the total genetic ablation of mGluR1 receptor in the SOD1^G93A^ mouse model produced a very serious ataxic and detrimental phenotype (unpublished results), thus excluding possible therapeutic approaches using mGluR1 pharmacological antagonists. Then, we tested the effect of the chronic oral administration of the mGluR5 NAM CTEP [311]. Differing from the mGluR5 genetic ablation, the pharmacological treatment was started after symptom onset and maintained until the late symptomatic stage of the disease [311]. CTEP dose-dependently ameliorated the survival and clinical course in SOD1^G93A^ mice. Of relevance, paralleling the genetic studies, chronic treatment with CTEP also reduced astrogliosis and microglia proliferation in the spinal cords of SOD1^G93A^ mice, possibly contributing to the amelioration of the extracellular noxious milieu toward MNs, thus in turn reducing the disease severity.

Considering the dual role of microglia during ALS progression and the fact that blocking mGluR5 before or after disease onset, by genetic or pharmacological strategies, respectively, always ameliorated disease progression and reduced glial reactivity, uncertainty about the mixed effects of dampening mGluR5 in ALS arises. The negative modulation of group I mGluRs should differently affect astrocyte or microglia cells early in the pathology, when microglia probably possess an anti-inflammatory phenotype and astrocytes should start to be reactive, with respect to the late symptomatic stages, when both astrocytes and microglia are detrimental for MNs.

Studying microglia cells isolated acutely at specific stages of the disease from the spinal cords of SOD1^G93A^ mice partially lacking mGluR5 allowed us to gather interesting, still unpublished results. We analyzed the cell phenotype at the pre-symptomatic and late disease stages to characterize the cell shift during the disease progression in SOD1^G93A^ mice, highlighting possible age- and sex-related differences. We also verified the impact of the genetic ablation of mGluR5 on the microglia phenotype. As expected, the expression of numerous microglia activation markers changed during disease progression, depicting a unique ALS microglia signature with no particular evidence of sex differences. Of note, mGluR5 dampening seems to strongly modify the microglia’s bioenergetic metabolism by promoting aerobic metabolism and decreasing the antioxidant response.

**Figure 2:**
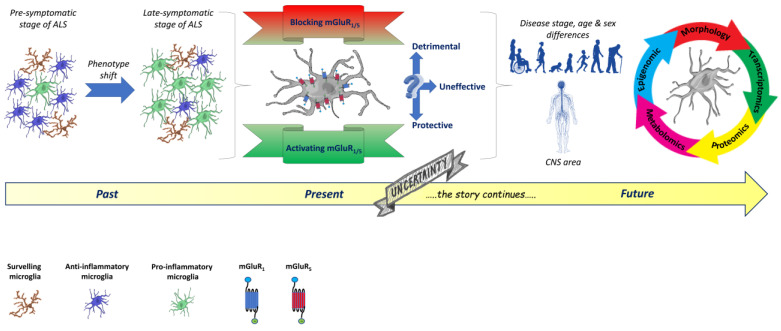
Past, present, and future perspectives to be further investigated in dissecting the role of mGluRs in the modulation of microglia phenotype changes during ALS progression. Past. Microglia phenotypes in ALS are mainly characterized by their different morphologies, depicting an unbalanced M1/M2 polarization ratio. During the early non-symptomatic stage of ALS, in response to different CNS microenvironmental signals, microglia cells acquire an anti-inflammatory phenotype, characterized by short and poorly branched processes and defined as M2, involved in the production of different protective effectors. The complex interplay between pathological signals from various cell types triggers a precocious microglia shift that acquires a detrimental pro-inflammatory phenotype, characterized by large cell bodies with short and dense processes, defined as M1, involving the production of several toxic factors. Present. During ALS progression, microglia cells do not undergo a stage-dependent discrete transition, but, instead, they show the coexistence of many different phenotypes with peculiar functional profiles, thus depicting a unique ALS microglia signature. Group I mGluR NAM and PAM modulate the microglia phenotype, ameliorating the disease outcome in experimental models of ALS. However, their most effective application mode, and the optimal intervention time window, still need to be determined. Future. Due to the subtle modifications that the microglia phenotype undergoes during specific ALS stages and the uncertain role played by mGluRs, “multiomic” investigation strategies, such as transcriptomic, proteomic, metabolomic, and epigenomic, are needed to shed light on this very complex scenario. These new, powerful approaches will hopefully unveil further details of tissue specificity, age-related diversity, and sex-dependent responses to a specific mGluR-based therapeutic intervention aimed at modulating the microglia phenotype. The figure was partly generated using Servier Medical Art, provided by Servier, licensed under a Creative Commons Attribution 3.0 unreported license https://creativecommons.org/licenses/by/3.0/ (accessed on 19 January 2023).

The studies mentioned above, including our microglia-specific effects of group I mGluR modulation, are worth considering in the potential therapeutic application of mGluR5-targeted drugs to be exploited for ALS and other neurodegenerative diseases characterized by glial activation and neuroinflammatory features. Due to the subtle modifications that the microglia phenotype may undergo during specific ALS stages, and the uncertain role played by mGluRs, the only way to shed light on this complex scenario would be to expand the studies by exploiting more powerful and “omic” approaches, including transcriptomics, proteomics, metabolomics, and epigenomics, besides those adopted till now, as schematically proposed in Figure 2.

### 5.4. Microglia Characteristics Dictate the Therapeutic Strategies in ALS

The responses of individual cell types to diseases in the CNS should be considered when using mGluR agonists or antagonists for therapeutic interventions, and the different approaches should be tailored depending on the specific pathological condition, on the optimal timing for the maximal efficacy, and on the cell subtype that needs to be targeted.

This aspect is particularly true in ALS, where the microglial cell activation and neuroinflammatory state play a prominent role. Progress has been recently made because of the growing interest in glial cells, particularly in microglia, and the development of innovative approaches targeting these specific cells. However, microglia cell signaling remains enigmatic, and many aspects of their pathophysiology still need to be unveiled. Different therapeutic strategies have been applied to reduce the pro-inflammatory activity of microglia and counteract the progression of the disease. Unfortunately, these anti-inflammatory therapies have had only modest success. The non-univocal role played by microglia during the disease might explain this partial failure.

To emphasize the crucial role of the phenotype balance of microglia cells in ALS and the importance of efficacious modulation to obtain therapeutic effects, we report the paradigm of minocycline administration, an antibiotic belonging to the tetracycline class. When minocycline was administered early before the detrimental shift toward a neuroinflammatory phenotype, it prevented microglia activation in ALS [312,313,314]. Instead, when administered later during the progression of the pathology, when microglia had already acquired a neurotoxic phenotype, the drug failed to counteract the pathological symptoms and even increased microgliosis [315]. Consistently, inhibiting the microglial function becomes a sound strategy only when the different stages of microglia polarization are considered, targeting the harmful factors or promoting the anti-inflammatory phenotype. Although very challenging, the normalization of the microglia polarization balance, inhibiting the pro-inflammatory phenotype, and simultaneously boosting the activation of protective microglia cells, appears to be one of the most promising therapeutic perspectives in the treatment of ALS [316,317,318,319].

Neurotransmitters, growth factors, cytokines, and their pathways lead to microglia reactivity regulation, but, moving deeper into the brain region, metabolic pathways and environmental factors are still a crucial challenge. Moreover, the design of tools able to antagonize the molecular triggers underlying microglia activation in the optimal time window is critical to ALS. Thus, the main future challenge will be to reveal the multifaceted aspects of microglia during disease progression and to indicate the correct time window in which to intervene, considering the local environment, which also affects the specific responses of microglia or other surrounding cells, positively amplifying or worsening the targeted approach. For instance, the local permissive milieu created by microglia is fundamental to guaranteeing the success of different therapeutic strategies aimed at reducing demyelination [320]. The above considerations justify the need for multimodal actions to normalize altered processes directly involving microglia, and the surrounding cells. Moreover, the individuation of specific time windows related to disease progression and possible sex-related differences in ALS are still fundamental aspects to be considered for future research.

Group I mGluRs are potentially druggable targets for ALS treatment because they effectively counteract the downstream consequences of excessive glutamate and represent fine receptor modulators, thus avoiding the well-known side effects generated by drugs acting at the ionotropic glutamate receptors. Moreover, exploiting a combination of mGlu5 and mGlu1 receptor NAMs, which would reduce excitatory transmission and glia reactivity, and mGluR3 PAMs, able to induce the production of neurotrophic factors, could represent an even more intriguing opportunity to reduce MN degeneration and glia reactivity in ALS.

## 6. Conclusions

The pharmacological or genetic modulation of group I mGluRs expressed by microglia cells represents an attractive multipotential therapeutic strategy for acute traumatic and chronic neurodegenerative disorders. Although the literature has frequently investigated the roles of mGluRs and related therapeutic approaches focusing on neurons, other cell types, including astrocytes and microglia, express these receptors. Many neuroprotective strategies aimed at modulating the aberrant reactive state of these cells have highlighted microglia as a new target to improve clinical outcomes in different pathological conditions, particularly neurodegenerative diseases characterized by prominent neuroinflammatory hallmarks. Group I mGluR modulation reduces inflammation, excitotoxicity, necroptosis, and apoptotic cell death. In this review, we present, a comprehensive overview describing the multifaced effects obtained by modulating group I mGluRs expressed in microglia cells in several acute or chronic CNS pathological conditions, such as trauma, spinal cord injury, cognitive disorders, epilepsy, intracerebral hemorrhage, ischemic stroke, Alzheimer’s disease, and ALS for the first time.

The pipelines of many pharmaceutical companies consider different strategies based on the group I mGluR modulation in treating neurological diseases. Unfortunately, none of these experimental compounds passed the final step for approval. ALS represents a neurodegenerative disease with urgent unmet therapeutic needs; thus, the possibility of exploring new approaches to cure it, i.e., the pharmacological modulation of group I mGluRs to counteract the aberrant glial reactivity, is a very appealing strategy.

As depicted in the present review, the further collection of pre-clinical and clinical evidence will be essential to optimize group I mGluRs as multipotential targets to modulate the complex balance of microglia phenotypes in CNS disorders.

## Data Availability

Not applicable.
